# Line-wave waveguide engineering using Hermitian and non-Hermitian metasurfaces

**DOI:** 10.1038/s41598-024-56049-7

**Published:** 2024-03-08

**Authors:** Haddi Ahmadi, Zahra Ahmadi, Nasrin Razmjooei, Mohammad Pasdari-Kia, Amirmasood Bagheri, Hamed Saghaei, Kamalodin Arik, Homayoon Oraizi

**Affiliations:** 1 Department of Electrical Engineering, Sharif University of Technology, 11155-4365 Tehran, Iran; 2 Department of Electrical Engineering, Tarbiat Modares University, 197-14115 Tehran, Iran; 3 Department of Electrical Engineering, University of Texas at Arlington, Arlington, 76019 USA; 4 Department of Electrical Engineering, Shahrekord Branch, Islamic Azad University, 8813733395 Shahrekord, Iran; 5 Department of Electrical Engineering, Iran University of Science and Technology, 1684613114 Tehran, Iran

**Keywords:** Line waves, Non-Hermitian line waves, Metasurfaces, Dual-band Waveguide, Graphene, Optics and photonics, Fibre optics and optical communications

## Abstract

Line waves (LWs) refer to confined edge modes that propagate along the interface of dual electromagnetic metasurfaces while maintaining mirror reflection symmetries. Previous research has both theoretically and experimentally investigated these waves, revealing their presence in the microwave and terahertz frequency ranges. In addition, a comprehensive exploration has been conducted on the implementation of non-Hermitian LWs by establishing the parity-time symmetry. This study introduces a cutting-edge dual-band line-wave waveguide, enabling the realization of LWs within the terahertz and infrared spectrums. Our work is centered around analyzing the functionalities of existing applications of LWs within a specific field. In addition, a novel non-Hermitian platform is proposed. We address feasible practical implementations of non-Hermitian LWs by placing a graphene-based metasurface on an epsilon-near-zero material. This study delves into the advantages of the proposed framework compared to previously examined structures, involving both analytical and numerical examinations of how these waves propagate and the underlying physical mechanisms.

## Introduction

Metasurfaces are artificially textured surfaces, possessing an electrical thickness much smaller than the wavelength^1^. They are crafted to manipulate electromagnetic wavefronts, amplitude, phase, and polarization^2^. In addition to empowering unparalleled control over reflection and transmission characteristics, the recently formed domain of flat optics has offered new insights into managing surface waves. This advancement, coupled with the transformative arrival of two-dimensional (2-D) materials, has sparked the adaptation of various captivating ideas from volumetric metamaterials into planar formats. These concepts encompass diverse elements such as hyperbolic propagation and transformation optics^3–5^.

More recently, there has been a surge of theoretical and experimental studies unveiling a new kind of EM mode. This wave propagates along an abrupt transition between metasurfaces distinguished by dual (capacitive/inductive) surface reactances. These waves retain the typical out-of-plane confinement characteristic of conventional surface waves on reactive metasurfaces. However, they also exhibit in-plane confinement along the transition, allowing them to transport energy along a one-dimensional path. As a result, they are termed “line waves” in the following discussion, although an alternative description such as “one-dimensional waves” has also been utilized^6–13^.

The implementation of Hermitian LWs, in which lossy impedance surfaces, generally speaking, are used, was first performed in the microwave regime^6^. Nevertheless, this implementation is constrained to a single frequency in the terahertz range, resulting in undefined bandwidth for LWs at higher frequencies^7^. In addition, LWs are found to be supported by non-Hermitian metasurfaces satisfying the parity-time symmetry. In these systems, the interface between two metasurfaces depends on the distinction in resistance (between gain and loss) rather than reactance (between capacitance and inductance)^14,15^. In this scenario, in contrast to traditional LWs, the fundamental method of localization does not depend on the EM duality; instead, it is based on the parity-time symmetry^16–20^. An intriguing aspect of employing graphene for LW implementation lies in the ability to actively manipulate the phase, intensity, and guided wavelength of the mode by adjusting surface impedance values along the interface line. Furthermore, in comparison to conventional edge modes, LWs benefit from a distinct advantage in terms of singular field enhancement. This characteristic, where infinite energy is concentrated along a line, represents another crucial aspect of these modes^21–25^.

In this context, we introduce a graphene patch design to present LWs in the terahertz and infrared ranges. While the implementation of LWs is typically limited to the terahertz range, we expand the operational range of these waves into the infrared domain by designing a dual-band line-wave waveguide. Since the classical metal conductivity model is no longer valid at higher frequencies, we use a graphene-based metasurface in an effort to implement the structure. Our designs consist of a graphene metasurface positioned atop two distinct substrates. One substrate is an ENZ substrate, which is a type of material with a very low effective permittivity close to zero, while the other substrate is a standard material with a typical permittivity value. Utilizing an epsilon-near-zero (ENZ) substrate transforms a passive metasurface into an active one. Our approach successfully meets both dual reactive impedances (inductive and capacitive) and impedance featuring a uniform reactive component alongside resistance with positive and negative values (gain and loss). We utilize non-Hermitian impedance boundaries, satisfying parity-time symmetry, to create a planar waveguide that supports LWs.

## Results and discussion

**Figure 1:**
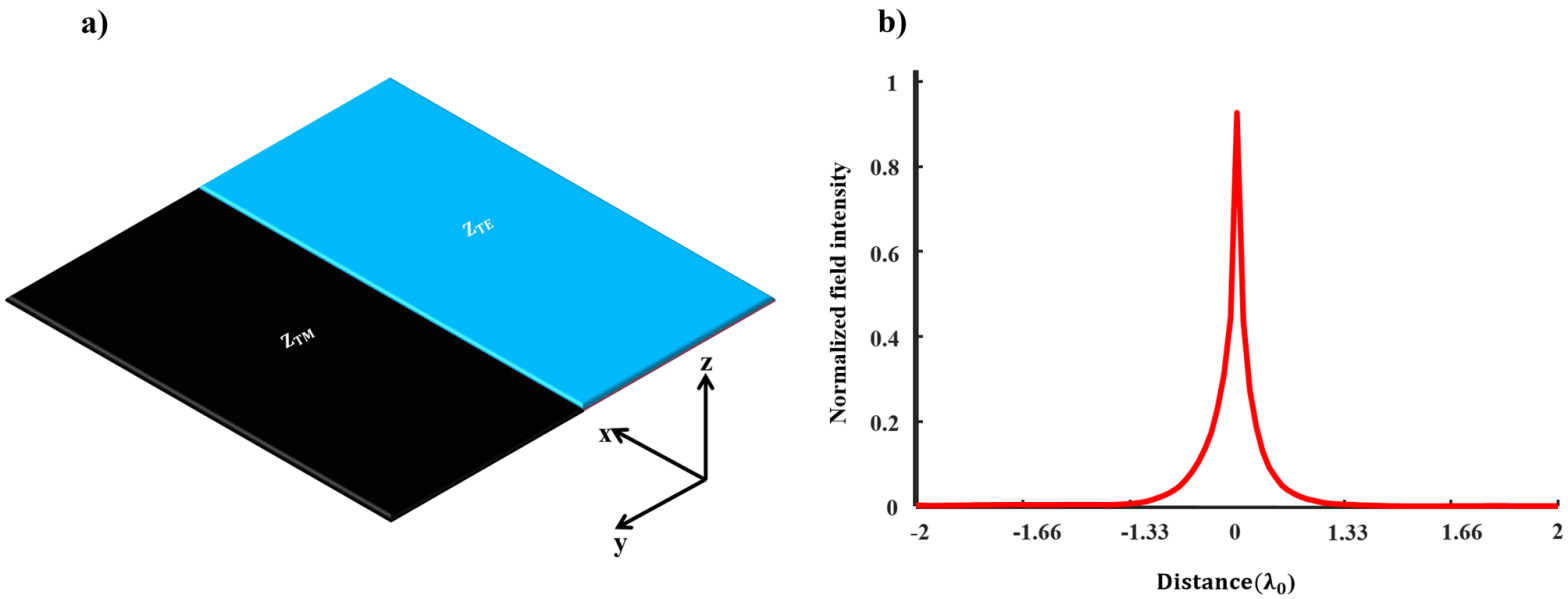
Unique characteristics of the LW. (**a**) A one-dimensional junction formed by interfacing dual impedance boundaries, and (**b**) singularity of the LW along the interface line of dual impedance surfaces. The LW decays exponentially in both transverse directions.

Figure 1a shows the schematic of interfaced impedance surfaces that can be considered as a layout designed to support LWs. The presence of two surface modes with perpendicular polarizations can be explained by homogeneous surface impedances in the following manner^6^

1 $$\begin{aligned} \begin{aligned} Z_{\textrm{s}}^{T M}&= R_{T M} + j \frac{\eta _{0}}{\zeta _{T M}} \end{aligned} \end{aligned}$$

2 $$\begin{aligned} \begin{aligned} Z_{\textrm{s}}^{T E}&= R_{T E} - j \eta _{0} \times \zeta _{T E} \end{aligned} \end{aligned}$$

where $\eta _{0}$ denotes the intrinsic impedance of free space, $\zeta$ is a real value, and *R*, representing the real component of surface impedance, can be either negative or positive. When $\zeta _{T M}=\zeta _{T E}$, inductive and capacitive impedance surfaces facilitate the propagation of symmetrical LWs. Since the LW with spin-up can exclusively move forward and the LW with spin-down can only go backward, the spin-momentum locking characteristic enforces the unidirectional nature of these states^26,27^. Indeed, according to the theorem of pseudospin states, two impedance surfaces preserving the EM duality satisfy mirror reflection symmetries. This leads to forming decoupled LWs known as pseudospin states^28^. In an ideal scenario, electromagnetic waves exhibit a singularity precisely at the interface line, as depicted in Fig. 1b. Additionally, surface impedances associated with non-matching electromagnetic responses support a quasi-line mode with a distribution resembling that of the edge modes.

**Figure 2:**
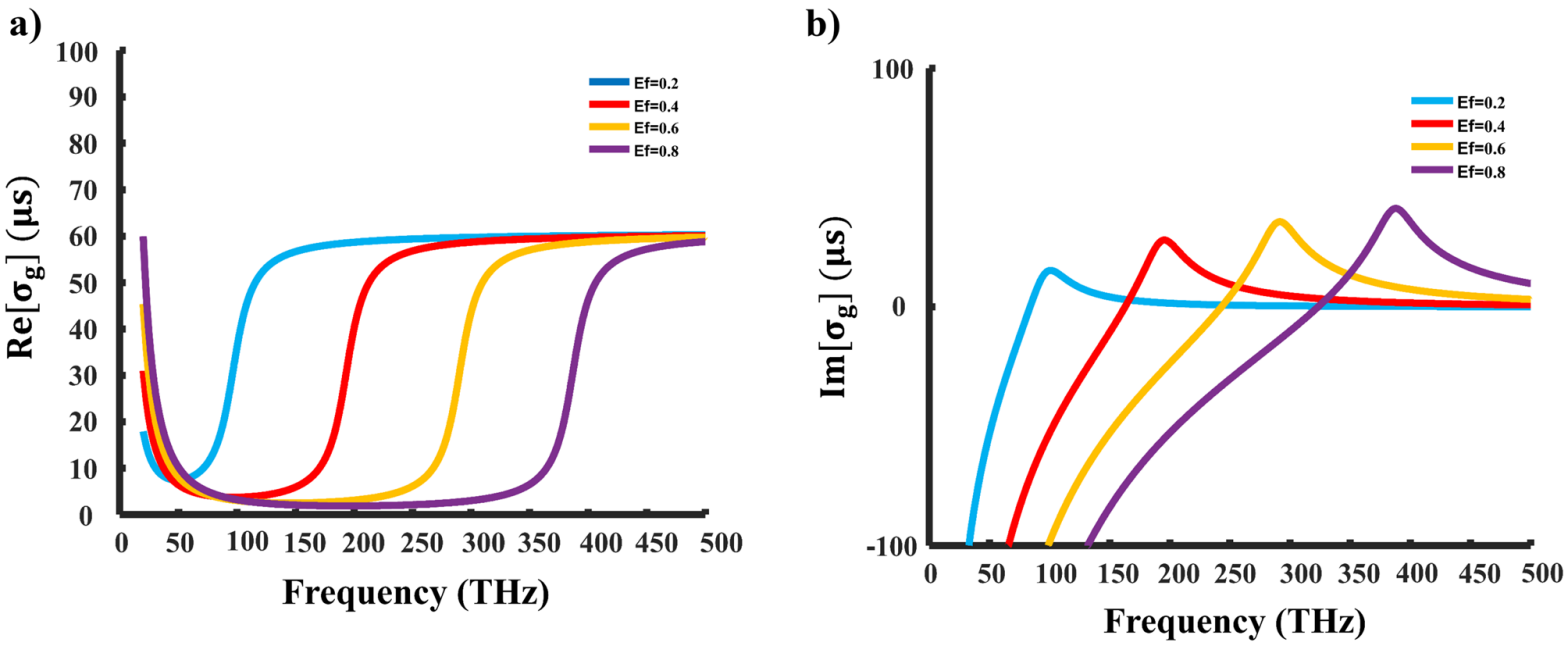
Graphene surface conductivity. (**a**) Real, and (**b**) imaginary parts of the graphene conductivity versus frequency and $E_{f}$ variations at room temperature.

Considering that graphene has the capability to support both TM and TE surface waves, one may utilize freestanding graphene to facilitate the propagation of LWs^29–31^. The equations provided below determine the dispersion characteristics of surface waves that find support on a graphene sheet^32^

3 $$\begin{aligned} \beta _{\text {TM}} = \frac{\omega }{c} \sqrt{1 - \left( \frac{2}{\sigma _{g} \eta _{0}}\right) ^{2}} \end{aligned}$$

4 $$\begin{aligned} \beta _{T E} = \frac{\omega }{c} \sqrt{1 - \left( \frac{\sigma _{g} \eta _{0}}{2}\right) ^{2}} \end{aligned}$$

where $\omega$ is the angular frequency, *c* is the speed of light in vacuum and $\sigma _{g}$ is the surface conductivity of graphene that can be derived using the Kubo formula as^33^

5 $$\begin{aligned} \begin{aligned} \sigma _g\left( \omega , E_f, \tau , T\right)&= \sigma ^{\text{ intra } }\left( \omega , E_f, \tau , T\right) + \sigma ^{\text{ inter } }\left( \omega , E_f, \tau , T\right) \\ \sigma ^{\text{ intra } }&= \frac{-j e^2}{\pi \hbar ^2\left( \omega -j \tau ^{-1}\right) }\left\{ E_f+2 k_B T \ln \left[ \exp \left( \frac{-E_f}{k_B T}\right) +1\right] \right\} \\ \sigma ^{\text{ inter } }&= \frac{e^2}{4 \hbar }\left( \frac{1}{2}+\frac{1}{\pi } \arctan \left[ \frac{\omega \hbar -2 E_f}{2 k_B T}\right] \right. \\&\quad \left. +\frac{j}{2 \pi } \ln \left[ \frac{\left( \omega \hbar +2 E_f\right) ^2}{\left( \omega \hbar -2 E_f\right) ^2+\left( 2 k_B T\right) ^2}\right] \right) \end{aligned} \end{aligned}$$

Here, we have $E_{f}$ representing the Fermi energy, $\hbar$ as the reduced Planck constant, $\omega$ as the angular frequency, *e* as the charge of an electron, $T =300$ K for temperature, and $\tau =10^{-13}\, \mathrm {~s}$ as the relaxation time. Figure 2a shows the real part of graphene conductivity plotted versus frequency at different $E_{f}$ values. The actual value of $\sigma _{g}$ remains positive across all frequencies and $E_{f}$ values, as depicted in Fig. 2a. Following Eqs. (3), and (4), when $\sigma _{g}$ is real, and the expression under the radicals is negative, both $\beta _{T M}$ and $\beta _{T E}$ become purely imaginary, indicating the propagation of evanescent surface modes. In essence, real values of $\sigma _{g}$ influence the dissipation loss of waves that are supported by the graphene sheet.

The imaginary component of $\sigma _{g}$ can change its sign when $E_{f}$ is switched from negative to positive or vice versa, as depicted in Fig. 2b. Consequently, the graphene structure has the capability to support either TE or TM surface waves depending on the value of $E_{f}$. To illustrate, we consider the propagation of surface plasmons in graphene at a frequency of $50 \textrm{THz}$. When $E_{f}=0.4$, the graphene surface conductivity becomes $\sigma _{g}=6.45 \times 10^{-6}-j 1.39 \times 10^{-4}$, indicating the propagation of TM surface waves in graphene. In this scenario, the normalized propagation constant is ${\text {Re}}\left[ \beta / k_{0}\right] =37.94$, indicating strong confinement of the wave to the structure and a guided wavelength for TM surface wave propagation significantly shorter than the wavelength in free space, i.e., $\lambda _{\text {guided-TM}} \ll \lambda _{0}$.

At $E_{f}=0.1$, the graphene surface conductivity becomes $\sigma _{g}=3.41 \times 10^{-5}+\textrm{j} 1.95 \times 10^{-6}$. This corresponds to TE surface wave propagation with a normalized propagation constant of ${\text {Re}}\left[ \beta / k_{0}\right] =1$. Therefore, in this case, TE mode propagation in graphene closely resembles TEM mode propagation in free space, where the guided wavelength $\lambda _{\text{ guided-TE }}$ is approximately equal to the free-space wavelength $\lambda _{0}$. It is important to note that this equality pertains to phase velocity, while field distributions may differ. For the propagation of a LW in a structure, it is essential for the phase velocities of surface waves with TM and TE polarization to be equal. However, freestanding graphene fails to meet this criterion, rendering it incapable of guiding LWs^7^.

## Hermitian line waves

Since implementing LWs using freestanding graphene is not feasible, one approach to achieve these waves is by utilizing graphene-based metasurfaces. In the realm of infrared wavelengths, metallic metasurfaces are unsuitable for LW implementation due to the absence of a well-established classical model for metal conductivity in this range. To address this challenge, we propose employing a graphene patch. We present two structures for guiding LWs: a dual-band waveguide that can support LWs in the terahertz and infrared spectra, and a non-Hermitian platform created by placing a graphene-patch metasurface on an ENZ substrate. We provide a numerical analysis of LW propagation in both Hermitian and non-Hermitian metasurfaces. The implementation of LWs has been limited to the terahertz regime. However, our proposed design opens up the possibility of implementing LWs in the infrared range. Non-Hermitian behavior can be achieved by introducing gain and loss elements into the graphene patch, which is placed on the ENZ substrate. Our approach to implementing non-Hermitian LWs is rooted in the parity-time symmetry.

**Figure 3:**
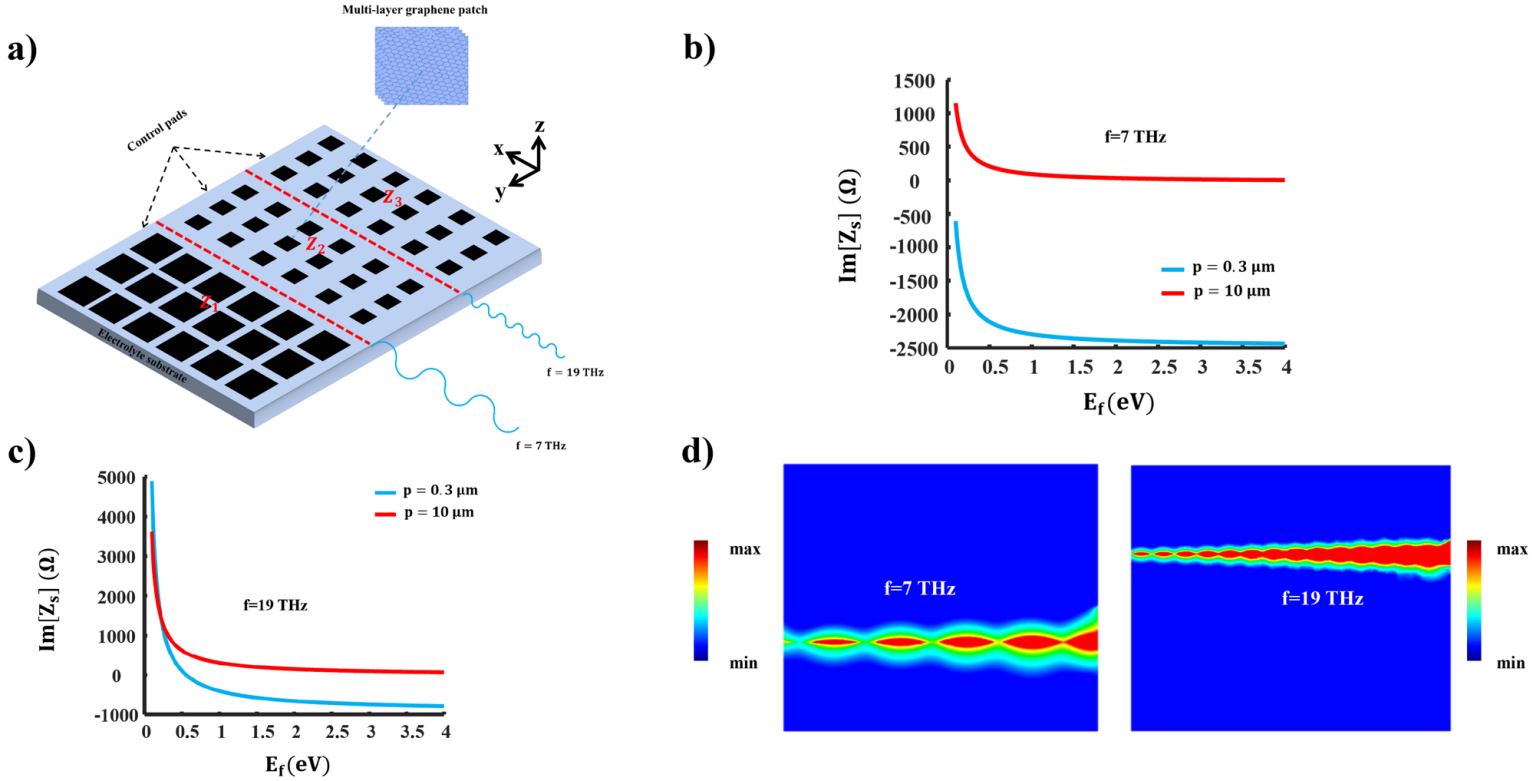
The proposed structure and its related characteristics. (**a**) A schematic that illustrates the proposed dual-band line-wave waveguide that utilizes a graphene-based metasurface, (**b**) the graph depicting the surface reactance of the structure at the frequency of $\omega / 2 \pi =7 \textrm{THz}$ versus chemical potential, (**c**) surface impedance of the graphene patch for different unit cell sizes at the frequency of $\omega / 2 \pi =19 \textrm{THz}$, and (**d**) electric field magnitude distribution at two different frequencies on top of the waveguide.

Figure 3a illustrates the schematic of our proposed structure, where graphene patch metasurfaces of different unit cell sizes are employed. The utilization of metasurfaces with different sizes aims to create impedances of both inductive and capacitive types at two distinct frequencies by altering $E_{f}$. To alter the Fermi energy levels of these unconnected graphene patches simultaneously, we propose the use of an extensive set of bias control pads positioned beneath the framework. This allows for the implementation of reconfigurable circuits so that LWs may be guided through optional pathways^7^. Although we consider two different channels for guiding LWs, this concept can be extended to encompass a more comprehensive, multi-channel scenario, allowing LWs to appear at multiple frequencies.

The suggested metasurface comprises regularly arranged subwavelength graphene patches within its structures. Just like other arrangements, these periodic arrays can be represented using equivalent impedance or equivalent surface conductivities in modeling. A closed-form expression is provided for equivalent impedance corresponding to the array of graphene patches which can be written as^34,35^

6 $$\begin{aligned} \begin{aligned} Z_{s}&= Z_{s1} + Z_{s2} \simeq \frac{p}{w \sigma } - j \frac{\pi }{2 \omega \varepsilon _{0} \varepsilon _{r} p \ln \left( \csc \left( \frac{\pi (p-w)}{2p}\right) \right) } \end{aligned} \end{aligned}$$

In this context, with *p* denoting the period, *w* indicating the patch width, and $\varepsilon _{r}$ set at 3.9 as the substrate’s dielectric constant, the electromagnetic behavior of the graphene patch can be seen as a series circuit comprising resistance (R), inductance (L), and capacitance (C). Generally speaking, and by way of illustration, we opt for $SiO_{2}$ as the substrate layer. The initial component, $Z_{s1}$, represents the series R–L resulting from the combination of the graphene surface impedance and geometric parameters. The second part, $Z_{s 2}$, corresponds to the capacitive response of the graphene impedance surface, which is dependent on the patch’s geometry and the background medium’s permittivity. It is important to note that the surface impedance of the graphene patch can be adjusted by altering the chemical potential. Nonetheless, there is a restriction on how much the chemical potential of graphene can be raised. To illustrate, when a gate voltage is applied to the graphene sheet through an electrostatic bias, it can elevate the carrier concentration, consequently affecting the values of $E_{f}$^36,37^.

Alternatively, the use of multilayer graphene sheets can lead to achieving higher Fermi energy. As a result, the total conductivity increases proportionally to the number of layers^7,38^. In this context, we examine a four-layer graphene patch metasurface as a means to attain higher levels of $E_{f}$. This, in turn, leads to a reduction in the real component of the surface impedance of the graphene patch, subsequently improving the propagation characteristics of the LW. Figure 3b illustrates the variation of the imaginary part of the graphene patch impedance in relation to changes in $E_{f}$ for different unit cell sizes at a frequency of $7 \textrm{THz}$. When $p=10\,\upmu$m and $w=8\,\upmu$m, it is possible to configure the impedance of the graphene patch to be inductive, whereas for $p=0.3\,\upmu$m and $w=0.15\,\upmu$m, the graphene patch’s impedance can be set as capacitive. Hence, achieving the duality condition for impedances at this frequency becomes readily feasible.

Likewise, in Fig. 3c, we depict the imaginary part of the graphene patch impedance at the frequency $\textrm{f}=19\, \textrm{THz}$. When $p=0.3\,\upmu$m and $w=0.15\,\upmu$m, the surface impedance can be either inductive or capacitive, contingent upon the appropriate choice of the $E_{f}$ value. Conversely, for $p=10\,\upmu$m and $w=8\,\upmu$m, the impedance can be tuned to become inductive. Following a similar approach, electromagnetic duality can be readily established at this frequency, enabling the creation of a dual-band line-wave waveguide by manipulating $E_{f}$ values at different frequencies. Figure 3d displays the electric field distribution of the LW at operational frequencies. At a frequency of $\textrm{f}=7 \textrm{THz}$, we have chosen $E_{f}$ values as follows: $E_{f1}=0.56$, $E_{f2}=E_{f3}=0.109$. At this frequency, the corresponding impedance values are $\mathrm {Z_1} =30+\textrm{j} 188.5$ and $\mathrm {Z_2}=\mathrm {Z_3} =277.6-\textrm{j} 754$. Note that $E_{f(i)}$ corresponds to $Z_{(i)}$. Similarly, at a frequency of $\textrm{f}=19 \textrm{THz}$, we set $E_{f}$ values to be $E_{f1}=1.26$, $E_{f2}=0.3$, and $E_{f3}=0.99$. The corresponding impedance values are $\mathrm {Z_1} =13.5+\textrm{i} 188.5$, $\mathrm {Z_2}=97.57+\textrm{i} 188.5$, and $\mathrm {Z_3}=27.2-\textrm{i} 754$. The proposed dual-band waveguide supports the propagation of two distinct frequency bands while maintaining a compact size. Furthermore, the line-wave waveguide can be tailored to support frequency bands that either overlap or do not overlap, depending on the specific requirements of the application.

## Line waves applications

LWs possess a range of distinctive characteristics that render them highly appealing for diverse applications. Among the notable advantages of line-wave waveguides is their one-dimensional nature, allowing for the creation of exceptionally compact waveguides for efficient energy confinement and transportation. This quality is particularly attractive in the realm of integrated photonics, where the demand for compact and efficient waveguides is pronounced. LWs also excel in mode confinement, rendering them valuable for applications involving interactions between light and matter, as well as chiral quantum processes. They can be effectively harnessed in the design of photonic devices aimed at manipulating the spin or polarization of light. Moreover, the capability to actively control LWs within graphene-based metasurfaces holds the potential for developing reconfigurable integrated circuits. These circuits could be dynamically adjusted to perform various functions, promising greater flexibility and efficiency in electronic devices^6,7^.

**Figure 4:**
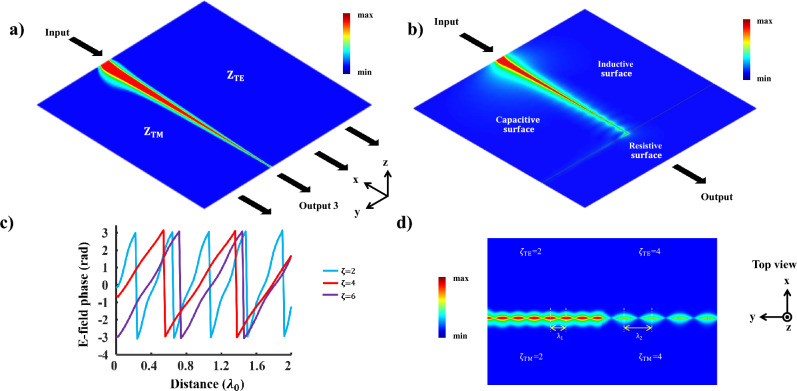
Illustration of possible applications of the LW. (**a**) A possible implementation of optical circuits such as a demultiplexer, and (**b**) a Not gate, (**c**) phase shift of the LW that corresponds to surface impedance varations across the interface line, and (**d**) control of the intensity and guided wavelength of the LW using $\zeta$ variation.

LWs present a promising foundation for signal processing applications, including the realization of logic gates operating in the infrared spectrum. The proposed configuration has the potential to be converted into a versatile waveguide by employing a substantial array of bias control pads positioned beneath the structure. This adaptability offers extensive circuit functionality and the capability to employ it for implementing logic gates. Figure 4a illustrates the deployment of a demultiplexer utilizing the line-wave waveguide. The surface impedance of the graphene patch is contingent on the Fermi energy, thus allowing for adjustment to be inductive, capacitive, or purely ohmic. The distinctive characteristic of the graphene patch presents an exceptional opportunity to guide LWs along different pathways by manipulating the $E_f$ values. This capability can be leveraged for LW switching, enabling the creation of adaptable waveguide networks. To realize a LW-based demultiplexer, it is possible to redirect the waveguide input toward the desired output. The selection of the desired output is accomplished through addressing, achieved by regulating $E_{f}$. In fact, the $E_{f}$ values serve as the selection lines for the 1-to-4 demultiplexer. For instance, by controlling the impedance surface values, the input can be directed to output 3.

Similarly, this configuration can be utilized to create a NOT gate. Since purely ohmic impedance surfaces lack the capacity to support surface waves, they can serve as a filtering component. This sets the stage for the implementation of a NOT gate, as depicted in Fig. 4b. This functionality hinges on the alteration of impedance surfaces through the adjustment of $E_{f}$. By modifying $E_{f}$, it becomes feasible to obstruct the wave’s propagation towards the output path. Consequently, the structure functions as an inverter, converting a logic level of 1 to 0. Conversely, to convert a logic level of 0 to 1, a control signal can be employed. In this scenario, when the waveguide input is 0, the output can be set to the logic level of 1^39^.

It is worth noting that, similar to gradient metasurfaces, graphene patches offer the potential to finely manipulate the phase, intensity, and propagation characteristics of electromagnetic fields. Specifically, this enables precise phase modulation of electric fields in response to $\zeta$ variations, as depicted in Fig. 4c. This capability makes it feasible to design a straightforward phase shifter without the need for added complexity. Figure 4d illustrates how the electric field distribution of the LW varies at different $\zeta$ values. The LW exhibits greater field intensity at lower $\zeta$ values. Furthermore, as $\zeta$ increases, the guided wavelength of LWs also increases, whereas LWs with lower $\zeta$ values remain tightly confined to the interface line. This adjustability in the LW characteristics adds to its appeal for radiation-related applications. It should be noted that the distribution of the fields is calculated at the frequency of 7 THz.

## Non-Hermitian line waves

LWs have been observed when two complementary impedance surfaces converge to form a one-dimensional channel. In this arrangement, one of the surfaces must exhibit inductive characteristics, facilitating the propagation of transverse magnetic surface waves. Conversely, the other surface should display capacitive properties, enabling the support of transverse electric surface waves. Moreover, research has indicated that if non-Hermitian metasurfaces adhere to parity-time symmetry conditions, line waves emerge at the interface of non-complementary surfaces. Consequently, LWs can materialize at the junction of impedance surfaces that are exclusively inductive or capacitive, influenced by a gain and loss mechanism.

**Figure 5:**
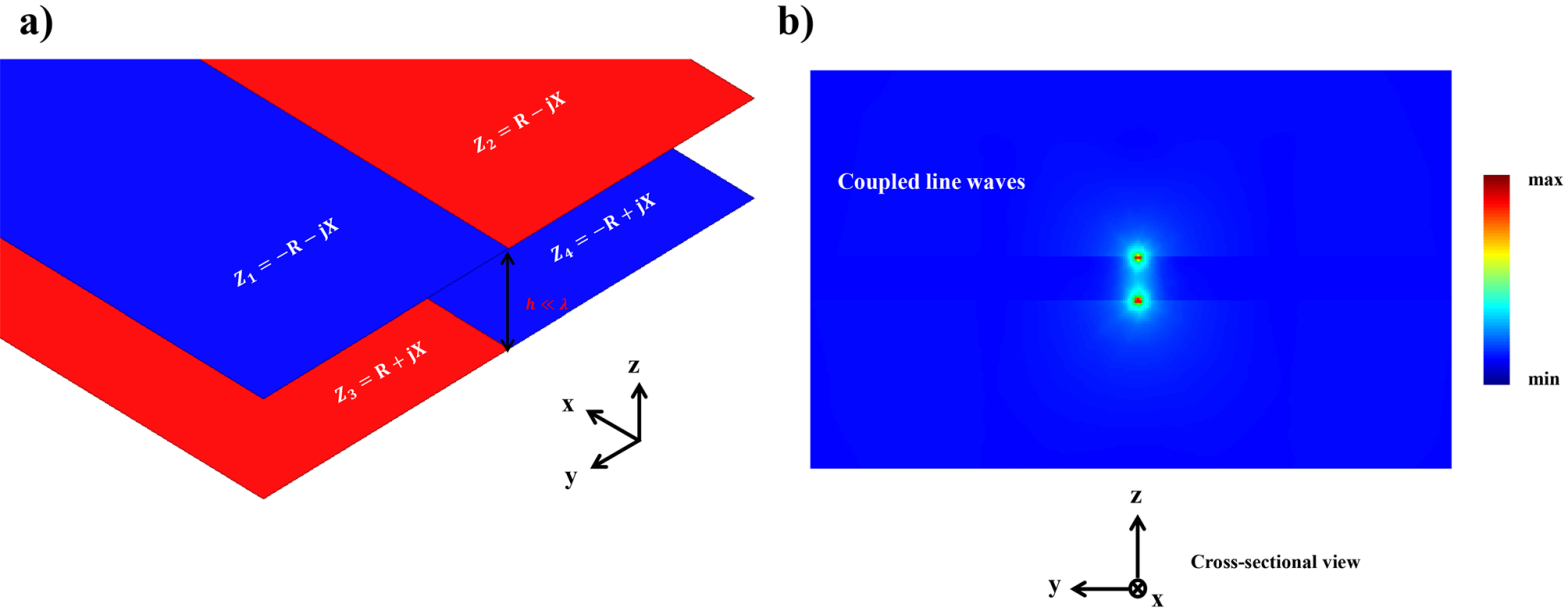
Non-Hermitian LWs. (**a**) The geometry of the problem, which involves a non-Hermitian impedance junction that can support coupled line waves, and (**b**) the distribution of the magnitude of the electric field of the LW at the cross-section of the waveguide.

It is worth noting that various arrangements of non-Hermitian metasurfaces can be explored to create novel waveguides capable of supporting LWs. Figure 5a illustrates the geometric layout of a junction formed by metasurfaces adhering to parity-time symmetry conditions, enabling the implementation of non-Hermitian LWs. In this setup, both non-Hermitian inductive and capacitive metasurfaces are employed to construct a parallel plate waveguide. The impedances composing the waveguide conform to the following parity-time symmetry condition

7 $$\begin{aligned} \begin{aligned} Z_1(x,y,z) = -Z_2(x,-y,z)^* \end{aligned} \end{aligned}$$

8 $$\begin{aligned} \begin{aligned} Z_3(x,y,z) = -Z_4(x,-y,z)^* \end{aligned} \end{aligned}$$

The impedance surfaces mentioned earlier, which create the waveguide boundaries, may not always exhibit mirror reflection symmetries. As a result, this waveguide does not facilitate the propagation of pseudospin states. Figure 5b shows the electric field distribution of LWs at the frequency of 10 THz. The impedance surfaces are spaced apart by a subwavelength distance, leading to the support of coupled LWs.

## Possible implementation of the non-Hermitian waveguide

A prevalent method for inducing non-Hermitian behavior involves introducing gain and loss effects through strategic positioning of gain materials, lossy dielectrics, or active elements within the meta-atoms^40–44^. These phenomena can be achieved at particular frequencies or across a broad spectrum, contingent upon the metasurface’s design. A practical realization of this concept could involve the utilization of photoexcited graphene^45^. Nevertheless, we employ a novel method to attain this outcome. It is important to note that when it comes to graphene-based metasurfaces on conventional substrates, higher chemical potential values are necessary to broaden the LWs’ operational range^7^.

**Figure 6:**
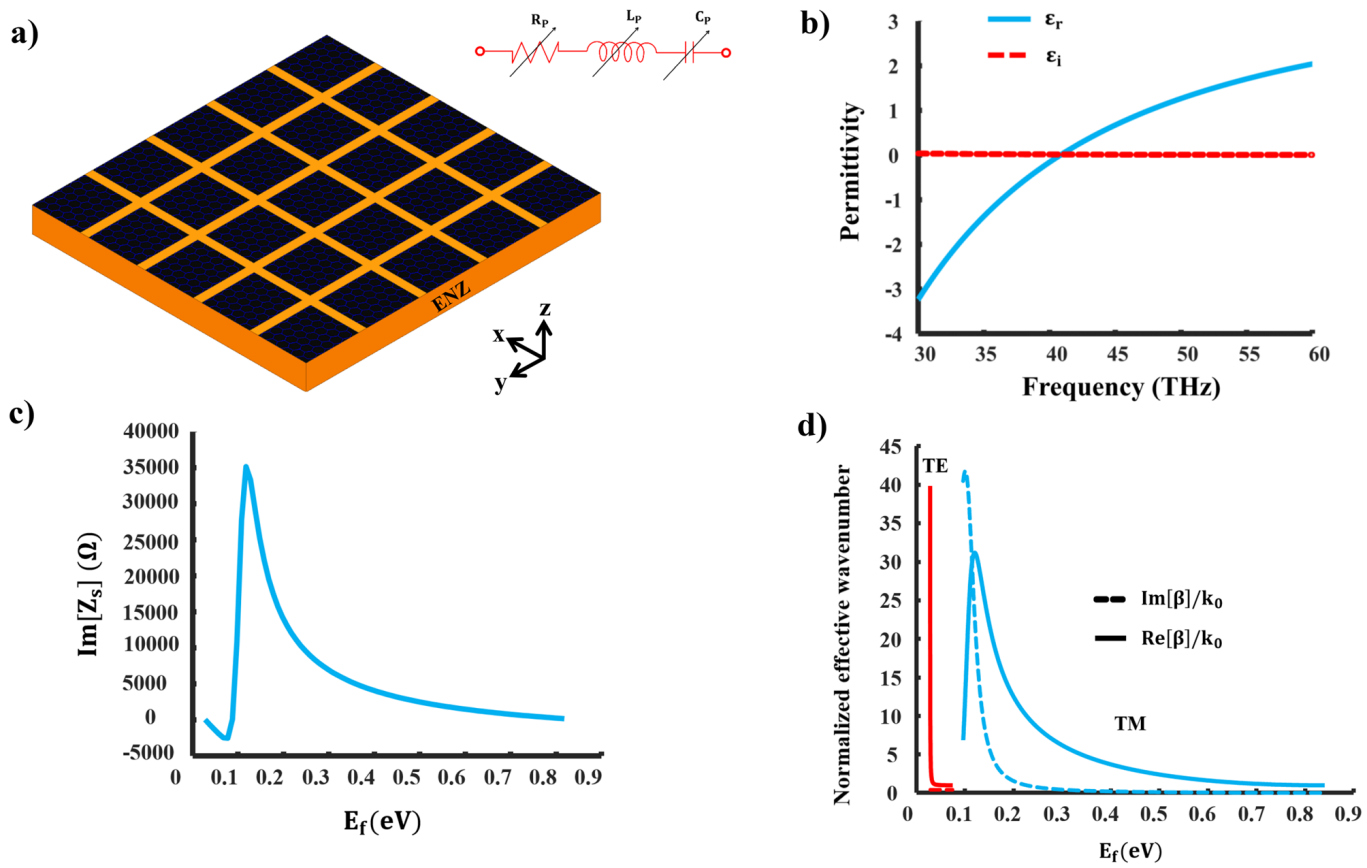
Characteristics and details of the proposed dual impedance surface. (**a**) Schematic of the proposed dual metasurface on an ENZ substrate and associated circuit model, (**b**) real and imaginary parts of aluminium-doped zinc oxide permittivity as a function of frequency, (**c**) the imaginary part of graphene patch impedance as a function of chemical potential at $f=42.2\,\textrm{THz}$, and (**d**) the associated normalized propagation constant of surface waves supported by the proposed structure.

To tackle this problem, our solution involves the use of a metasurface made of graphene patches, which demonstrates both capacitive and inductive electromagnetic responses. This metasurface is positioned on a material with epsilon-near-zero (ENZ) properties, as depicted in Fig. 6a. We opt for aluminum-doped zinc oxide as the ENZ substrate, and describe the complex permittivity of this material by applying the Drude oscillator model^46–48^

9 $$\begin{aligned} \begin{aligned} \varepsilon&= \varepsilon _{r} + j \varepsilon _{i} \\&= \varepsilon _{b} - \frac{\omega _{p}^{2}}{\left( \omega ^{2}+\gamma ^2\right) } + j \frac{\omega _{p}^{2} \gamma }{\left( \omega ^{2}+\gamma ^{2}\right) \omega } \end{aligned} \end{aligned}$$

where $\varepsilon _{b}=3.8$ represents the high-frequency permittivity, $\omega _{p}=0.5 \times 10^{15}$ corresponds to the plasma frequency, which is directly related to the concentration of free carriers, and $\gamma =0.1 \times 10^{13}$ denotes the Drude damping rate. These parameters collectively influence the ENZ frequency at which $\varepsilon _{r}$ reaches zero within the infrared range, as illustrated in Fig. 6b.

It is worth noting that the surface impedance of the graphene patch has a unique dual property (that is, it can either be inductive or capacitive). This sets it apart from subwavelength metallic patch/grid metasurfaces, which typically exhibit a predominantly reactive response. This dual characteristic of the graphene patch allows for the manipulation of the electromagnetic response of the structure by adjusting $E_{f}$ while keeping the frequency constant. Figure 6c provides insight into the surface impedance of the graphene patch at a frequency of $\textrm{f}=42.2\,\textrm{THz}$ with $\varepsilon =0.24+\textrm{j} 0.01$, $p=\lambda _0/10$, and $w=4\times \lambda _0/50$ in which $\lambda _0$ is the wavelength which corresponds to the operating frequency. When coupled with an ENZ substrate, the EM response of the graphene patch shifts from being capacitive at lower $E_{f}$ values to becoming inductive at higher $E_{f}$ values.

**Figure 7:**
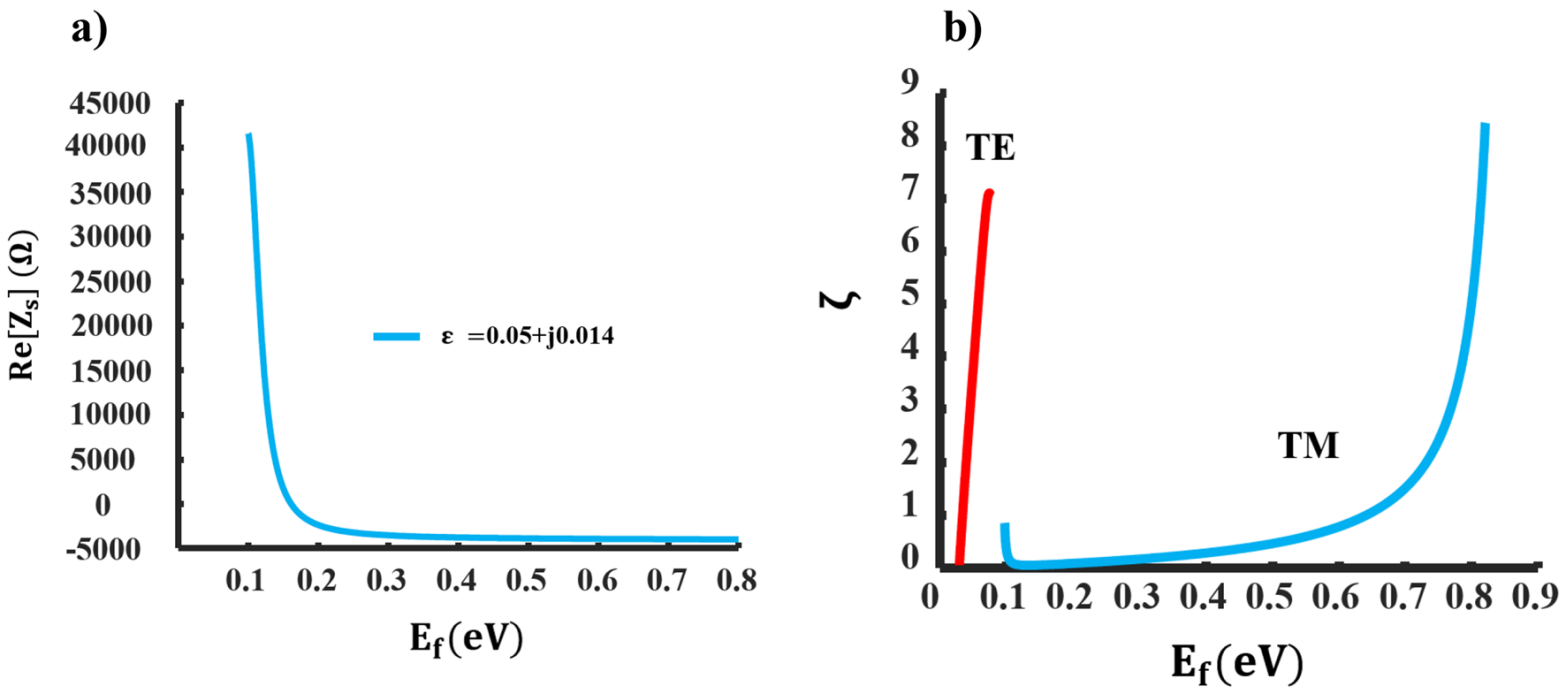
EM response of the graphene patch positioned on the ENZ substrate. (**a**) Gain effect achieved by properly adjusting of the $E_{f}$ value, and (**b**) $\zeta$ values of surface waves versus $E_f$.

The equations below can be employed to compute the dispersion relationships for the equivalent surface impedance of the proposed structure^7–15,49^

10 $$\begin{aligned} \begin{aligned} \beta _{TM} = \frac{\omega }{c} \sqrt{1 - \left( \frac{Z_{s}^{TM}}{\eta _{0}}\right) ^{2}} \end{aligned} \end{aligned}$$

11 $$\begin{aligned} \begin{aligned} \beta _{TE} = \frac{\omega }{c} \sqrt{1 - \left( \frac{\eta _{0}}{Z_{s}^{TE}}\right) ^{2}} \end{aligned} \end{aligned}$$

In Fig. 6d, we can observe the confinement factor of surface waves supported by the graphene patch at room temperature. For the mentioned parameters, within the range of $0.028 \le E_{f} \le 0.075$, a TE mode confinement factor ranging from $1 \le {\text {Re}}\left[ \beta / k_{0}\right] \le 39.85$ is obtained. However, as $E_{f}$ increases, a TM mode emerges. In this scenario, within the range of $0.096 \le E_{f} \le 0.84$, a confinement factor of $1.002 \le {\text {Re}}\left[ \beta / k_{0}\right] \le 31.2$ is achieved, and it is worth noting that a three-layer graphene patch is utilized on the TM side to compute the dispersion relation of the TM surface wave. The inclusion of a multi-layer graphene patch serves the purpose of minimizing dissipation losses. Additionally, the use of an ENZ substrate enables the implementation of LWs with low values of $E_{f}$.

In practice, achieving the condition of parity-time symmetry, which involves reversing the resistance, is a considerably difficult challenge. Instead, due to the fact that electrons and holes in optically pumped graphene have energy spectra without energy gaps, negative resistance occurs specifically in the terahertz frequency range^50,51^. Our suggested design opens the door to introducing the gain-loss phenomenon in the infrared spectrum. This facilitates the utilization of LWs by harnessing both duality and parity-time symmetries. Since the material that is used as an ENZ substrate has a complex permittivity, according to Eq. (6) the real part of surface impedance becomes negative at some specific frequencies. This means that the structure can be used in an attempt to implement waveguiding systems that support non-Hermitian LWs. Consequently, the proposed structure is a feasible way to attain the non-Hermitian behavior. If a complex-valued permittivity is considered as $\epsilon _r=Re(\epsilon _r)+Im(\epsilon _r)$, the real part of graphene patch surface impedance according to Eq. (6) can be written as follows

12 $$\begin{aligned} \begin{aligned} \text {Re}(Z_{s})&= \text {Re}(Z_{s1}) - \frac{\pi \text {Im}(\varepsilon _{r})}{2 \omega \varepsilon _{0} (\text {Re}(\varepsilon _{r})^2+\text {Im}(\varepsilon _{r})^2) p \ln \left( \csc \left( \frac{\pi (p-w)}{2p}\right) \right) } \end{aligned} \end{aligned}$$

According to Eq. (12), $Re(Z_{s})$ can be negative providing its first part is smaller than the second part. This can be achieved at some frequencies with a lossy ENZ substrate. Figure 7a illustrates the resistive component of the proposed impedance surface. A shift from positive resistance (indicating loss) to negative resistance (indicating gain) occurs at $\varepsilon =0.05$ $+\textrm{j} 0.014$ and a frequency of $41 \textrm{THz}$. In Fig. 7b, one can see the $\zeta$ values plotted against $E_{f}$. The maximum values of $\zeta$ are achieved with $\zeta _{T M} \le 8.43$ for a three-layer graphene metasurface and $\zeta _{T E} \le 7.03$ for a one-layer metasurface. These values are notably higher compared to the scenario where a conventional substrate is used, and their upper limit is substantially increased. Consequently, this leads to an expanded operational range for LWs by controlling the $\zeta$ values. It is critical to bear in mind that the proposed layout can be used to implement both Hermitian and non-Hermitian LWs.

## Eigenmode analysis

**Figure 8:**
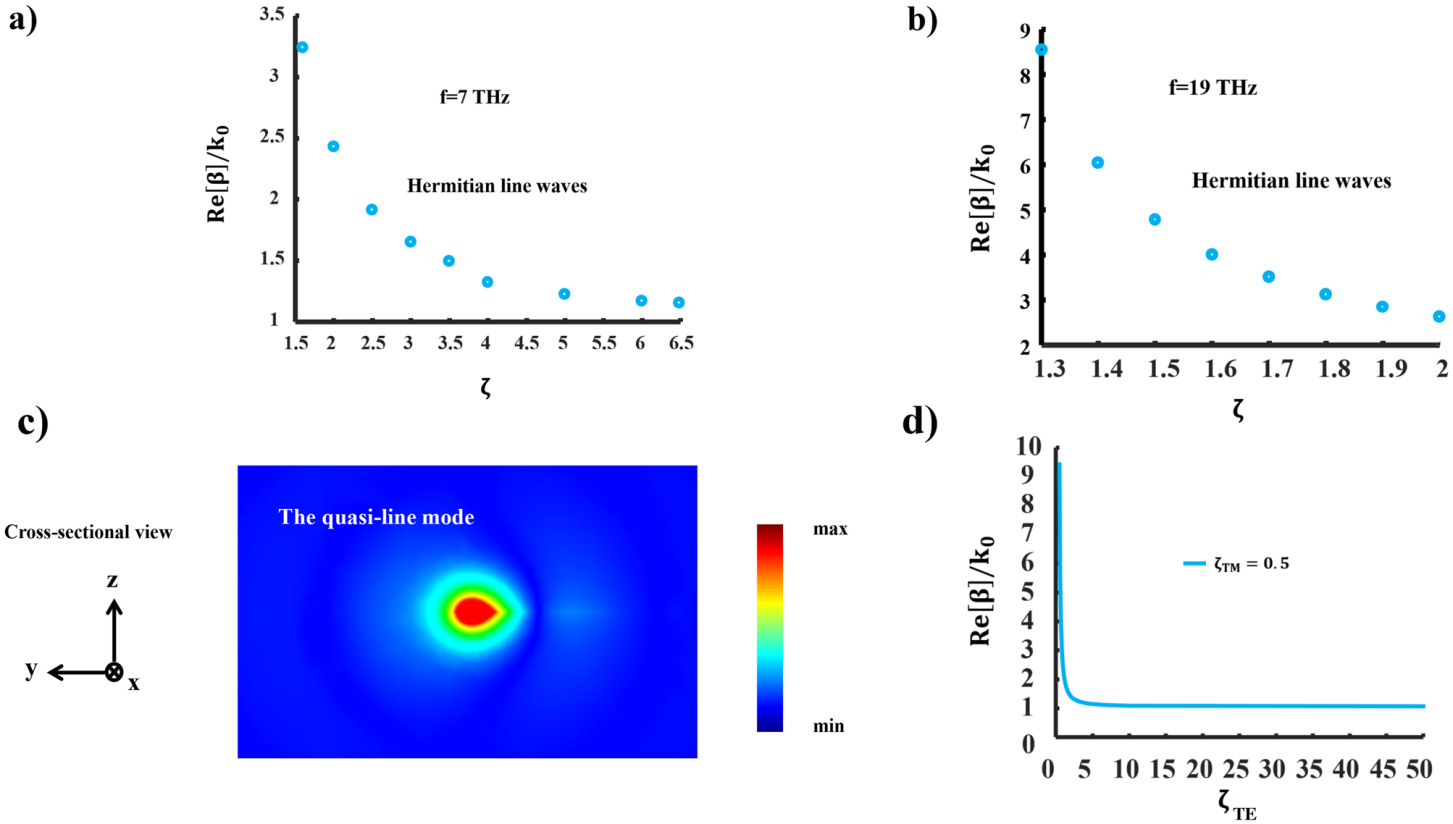
Eigen-mode analysis. (**a**) Dispersion characteristics of the line mode for the dual-band waveguide at the frequency of f= 7THz, (**b**) at the frequency of f = 19 THz, (**c**) E-field distribution of the quasi-line mode at the waveguide cross section, and (**d**) the dispersion diagram of the quasi-line mode.

Figure 8a illustrates the dispersion characteristics of the line wave in the dual-band waveguide at a frequency of 7 THz. To analyze the dispersion features of the line mode, we consider impedance surfaces with complex values. It is important to note that the $\zeta$ range at each frequency is determined by the values of the surface impedances. For $\zeta$ values ranging from 1.6 to 6.48, a confinement factor within the range of $1.18\le {\text {Re}}[\beta ] \le 3.42$ is achieved. The confinement factor decreases proportionally with an increase in $\zeta$, while for $\zeta \le 1.3$, there is an evanescent wave along the propagation direction, indicating that ${\text {Re}}[\beta ] \le$ ${\text {Im}}[\beta ]$. Conversely, $\zeta <1$ corresponds to the LWs cutoff, in accordance with observations.

Figure 8b, depicts the confinement factor of the line mode at a frequency of 19 THz. For $\zeta$ values ranging from 1.3 to 2, a higher confinement factor within the range of $2.62\le {\text {Re}}[\beta ] \le 8.56$ is achieved. However, there are limitations on the $\zeta$ values. It is worth noting that all limitations pertaining to the dispersion characteristics of the line mode are dictated by the real and imaginary parts of surface impedances. As discussed earlier, non-complementary metasurfaces support a quasi-line in which a portion of electromagnetic energy is restricted within the supporting metasurfaces. Consequently, this mode exhibits a striking similarity to well-known edge modes as shown in Fig. 8c. Lastly, Fig. 8d demonstrates the propagation characteristics of a quasi-line mode. This mode exhibits a confinement factor ranging from $1.05 \le {\text {Re}}[\beta ] / k_{0} \le 9.4$ for $\zeta$ values ranging from 0.2 to 50. Unlike the conventional line mode, a quasi-line mode exists for $\zeta <1$, provided that $\zeta _{TM} \times \zeta _{TE}>1$.

## Discussion

**Figure 9:**
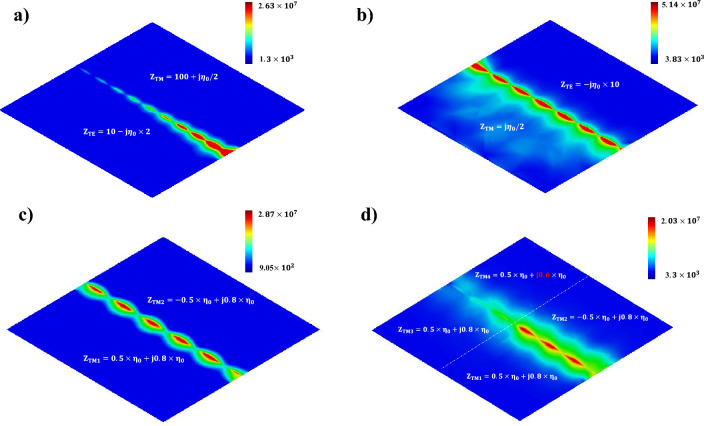
LWs at the interface of Hermitian, and non-Hermitian impedance boundaries. (**a**) LWs supported by dual impedance surfaces that are lossy. (**b**) Quasi-line waves at the interface of dual impedance surfaces having unequal $\zeta$ values. (**c**) LWs supported by non-Hermitian inductive impedance surfaces. (**d**) LWs filtering caused by introducing a defect in the system.

To implement non-Hermitian line-wave waveguides in practice, it is crucial that the parity-time symmetry should be satisfied. According to Eqs. (7) and (8), the parity-time symmetry condition necessitates complete equality in the imaginary part of surface impedances, and the absolute value of the real part of surface impedances should be identical, with one surface having negative resistance and the other positive resistance. As previously discussed, in the case of LWs guided by Hermitian surfaces, inequality in the values of $\zeta$ leads to the appearance of the quasi-line wave. Conversely, unequal real parts of impedances in Hermitian LWs do not impact the mode symmetry and do not hinder the propagation of LWs. The practical implementation of Hermitian LWs over a wide range of $\zeta$ values can be easily achieved. However, implementing quasi-line waves using non-Hermitian surfaces is not feasible due to the violation of the parity-time symmetry condition. Particularly, this feature holds true for the non-Hermitian inductive impedance surfaces. It is worth noting that unequal resistance values also violate the parity-time symmetry condition. Indeed, precise compliance with this condition results in the appearance of non-Hermitian LWs, imposing significant limitations on the practical implementation of non-Hermitian line modes within specific frequency ranges. For better comparison, Fig. 9a illustrates the distribution of Hermitian LWs guided by lossy dual surfaces, enabling symmetrical line mode guiding. The field distribution, more commonly observed in the propagation of LWs along the interface of graphene-based metasurfaces, has been calculated for $Z_{TE}=10-j\eta _0\times 2$, and $Z_{TM}=100+j\eta _0/2$ at the frequency of 10 THz.

Conversely, as depicted in Fig. 9b, non-complementary dual-impedance surfaces failing to satisfy the $Im(Z_{TM})\times Im(Z_{TE})=\eta _0 ^2$ relationship support quasi-line waves. The field distribution is calculated for $Z_{TE}=-j\eta _0\times 10$, and $Z_{TM}=j\eta _0/2$ at the same frequency. In this scenario unequal selection of $\zeta$ values results in the greater amplitude of EM fields on the TM side.

For a clearer comparison, Fig. 9c showcases the distribution of non-Hermitian LW modes for $Z_{TM1}=0.5\times \eta _0+j0.8\times \eta _0$, and $Z_{TM2}=-0.5\times \eta _0+j0.8\times \eta _0$, where the parity-time symmetry relation is basically satisfied, resulting in a symmetric field distribution. As previously discussed, the non-adherence to parity-time symmetry hampers the propagation of non-Hermitian LWs. For instance, Fig. 9d displays the propagation of LWs in a non-Hermitian waveguide, where a disturbance is created towards the end of the waveguide by selectively choosing impedance values as $Z_{TM1}=0.5\times \eta _0+j0.8\times \eta _0$, $Z_{TM2}=-0.5\times \eta _0+j0.8\times \eta _0$, $Z_{TM2}=0.5\times \eta _0+j0.8\times \eta _0$, and $Z_{TM4}=-0.5\times \eta _0+j0.6\times \eta _0$. Obviously, this disturbance prevents wave propagation. It is important to note that applying this disturbance to the real part of impedances would also inhibit the appearance of non-Hermitian LWs. Therefore, practical limitations arise concerning non-Hermitian LWs when considering the complex requirement of parity-time symmetry.

**Table 1:** Comparison of key parameters of reported waveguides.

Characteristic	This work	Reference^7^	Reference^14^
Ability to support quasi-line waves	True	True	N/A
Structure is a one-way waveguide	True	True	N/A
Structure is a dual-band waveguide	True	N/A	N/A
Simultaneous guidance of Hermitian and non-Hermitian line modes	True	N/A	N/A
Feasibility for implementing reconfigurable circuits	True	True	N/A

Our proposed waveguiding structure offers several distinctive advantages over previously studied structures. For instance, the proposed Hermitian line-wave waveguide supports one-way modes called “pseudospin states”^28^. Table 1 presents a comprehensive comparison between the characteristics of the proposed waveguides and the structural configurations investigated in the literature (references^7,14^. This table meticulously outlines and contrasts various structural features, offering a detailed overview and analysis of the similarities and differences observed among these different waveguide designs. In the tabe, “N/A” stands for “Not Applicable”.

## Conclusion

In summary, we introduce a graphene patch with a unique dual capacitive–inductive property, offering the potential for both Hermitian and non-Hermitian line-wave waveguides. We explore various applications for these waveguides and demonstrate that placing the graphene patch on an ENZ substrate facilitates the creation of waveguides with low $E_{f}$ values. By adjusting $E_{f}$ and the number of layers in the multilayer graphene structure, we can control both positive and negative resistance values in the structure, thereby influencing the transition characteristics of line waves. We also highlight how modifications to $\zeta$ can impact line wave intensity, phase, and confinement, suggesting possibilities for reconfigurable circuit development and controllable logic gates. Furthermore, we introduce different planar waveguide configurations capable of supporting line waves, including a dual-band waveguide, and a parallel plate waveguide that guides coupled line waves.

## Data Availability

The data that support the findings of this study are available from Haddi Ahmadi upon reasonable request.
